# Single-leg stance on a challenging surface can enhance cortical activation in the right hemisphere – A case study

**DOI:** 10.1016/j.heliyon.2023.e13628

**Published:** 2023-02-10

**Authors:** Haroon Khan, Nauman Khalid Qureshi, Anis Yazidi, Håvard Engell, Peyman Mirtaheri

**Affiliations:** a Department of Mechanical, Electronics and Chemical Engineering, OsloMet – Oslo Metropolitan University, Pilestredet 46, 0167 Oslo, Norway; b Institute of Technology for Architecture (ITA), ETH Zürich, Switzerland; c Department of Computer Science, Oslo Metropolitan University, Pilestredet 46, 0167 Oslo, Norway; d GAITLINE AS, Maridalsveien 300, 0872 Oslo, Norway

**Keywords:** Functional near-infrared spectroscopy (fNIRS), Single-leg stance (SLS), General linear model (GLM), Cortical activation, Alpine ski, Dual-leg stance (DLS)

## Abstract

Maintaining body balance, whether static or dynamic, is critical in performing everyday activities and developing and optimizing basic motor skills. This study investigates how a professional alpine skier's brain activates on the contralateral side during a single-leg stance. Continuous-wave functional near-infrared spectroscopy (fNIRS) signals were recorded with sixteen sources and detectors over the motor cortex to investigate brain hemodynamics. Three different tasks were performed: barefooted walk (BFW), right-leg stance (RLS), and left-leg stance (LLS). The signal processing pipeline includes channel rejection, the conversation of raw intensities into hemoglobin concentration changes using modified Beer-Lambert law, baseline zero-adjustments, z-normalization, and temporal filtration. The hemodynamic brain signal was estimated using a general linear model with a 2-gamma function. Measured activations (*t*-values) with *p*-value <0.05 were only considered as statistically significant active channels. Compared to all other conditions, BFW has the lowest brain activation. LLS is associated with more contralateral brain activation than RLS. During LLS, higher brain activation was observed across all brain regions. The right hemisphere has comparatively more activated regions-of-interest. Higher ΔHbO demands in the dorsolateral prefrontal, pre-motor, supplementary motor cortex, and primary motor cortex were observed in the right hemisphere relative to the left which explains higher energy demands for balancing during LLS. Broca's temporal lobe was also activated during both LLS and RLS. Comparing the results with BFW– which is considered the most realistic walking condition–, it is concluded that higher demands of ΔHbO predict higher motor control demands for balancing. The participant struggled with balance during the LLS, showing higher ΔHbO in both hemispheres compared to two other conditions, which indicates the higher requirement for motor control to maintain balance. A post-physiotherapy exercise program is expected to improve balance during LLS, leading to fewer changes to ΔHbO.

## 1 Introduction

Many balance training and rehabilitation programs use the single-leg stance (SLS) for various balance disorders investigations [1]. Sensory systems, muscular activations, and passive dynamics (i.e., ligaments and joints) must coordinate with the central nervous system for balance and control [2]. About forty percent of the gait cycle is spent in a single-leg stance, and it's also an essential posture in any case, such as walking stairs [3]. Walking is a dynamic movement compared to the SLS assessment, but the stabilization mechanism is essentially the same. Research has previously explored the distribution of plantar pressure during an SLS; however, the efficiency of postural control processes during a one-leg stance may also depend on complex cortical control, which is poorly understood [4]. SLS is not only important for postural stability but also an important test for brain health [5]. According to the latter study, people who appear unbalanced on one leg should receive increased attention as this may indicate an increased risk for brain disease and cognitive decline. Noncontact lower extremity injuries may be predicted by the transition from double-leg stance (DLS) to SLS [6]. It is important to understand the overall activation of the cortex and the lateralization of motor control for complex tasks like SLS. The left hemisphere is generally dominant, giving the right side of the body better neuromuscular control. However, according to most balancing tests, such as the center of pressure (COP) displacement, standing on the dominant leg compared to the non-dominant leg did not lead to statistically significant differences in various balance measures and even in electromyography (EMG) [7]. Each leg must be used in any training condition to improve performance. Working more on the dominant leg will likely improve the person's preferred side in sport.

In conventional neuroimaging, it is rare to explore the dynamic balance control due to the static nature of the hardware. However, the advancement in two of the most prominent neuroimaging modalities, i.e., electroencephalography (EEG) and functional near-infrared spectroscopy (fNIRS) have enabled the possibility of mobile neuroimaging to investigate dynamics balance control. Yet, very few studies are still available [8], [9]. The reason might be the non-availability of mobile neuroimaging technologies in the past. Due to the portability of neuroimaging and ease of use, fNIRS is gaining popularity in brain-computer interface (BCI) applications, particularly in rehabilitation [10], [11], [12], [13]. A number of the balance-challenging tasks were shown to activate the prefrontal cortex (PFC) [14], premotor cortex (PMC), and supplementary motor area (SMA) [8]. An SLS modulates cortical activity based on the stability of the leg and the surface [15]. In healthy adults, an enhanced activation was observed in SMA, pre and post-central gyrus during standing on a balance board [16] compared to DLS. In another study, Luks et al. [17] pointed out that the dorsolateral prefrontal cortex (DLPFC) may be involved in maintaining postural control through attention allocation.

During SLS, there is little understanding of contralateral cerebral hemoglobin oxygenation. This case study aims to explain the relationship between cortical activation in the right (RLS) and left-leg stance (LLS) in the best alpine skier. Furthermore, the contralateral changes in the brain's oxygenated (ΔHbO) on either side of the hemisphere correspond to RLS and LLS. Therefore, the study hypothesized that better balance control corresponds to less brain activation. An increase in postural instability and task difficulty increases cortical activation and connectivity [18]. Researchers have found that postural balance activates the prefrontal, premotor, supplementary motor, and parietal cortical networks in healthy subjects and post-stroke patients [19]. Also, observing the effect of particular physiotherapy exercises on brain activation is crucial to investigate in later stages. The remainder of the paper is organized as follows. Section 2 describes the methodology. In section 3, we provide the results and discuss them, while Section 4 concludes the paper.

## 2 Methodology

### 2.1 Participant

The participant in the experiment is a world-class alpine skier with excellent physical health and better balance and motor control than an average human. Comparatively, the participant felt less balance and control of the left leg, which was intriguing to observe the effect on the contralateral brain motor control. Explicit consent was obtained from the participant for participation and publication. The work is approved by the Norwegian center for research data (reference no. 751430) and Regional committees for medical and health research ethics (reference no. 322236), Norway. The experiment was conducted according to the latest declaration of Helsinki.

### 2.2 Experimental setup, paradigm, instruction, and observation during experiment

**Figure 1:**
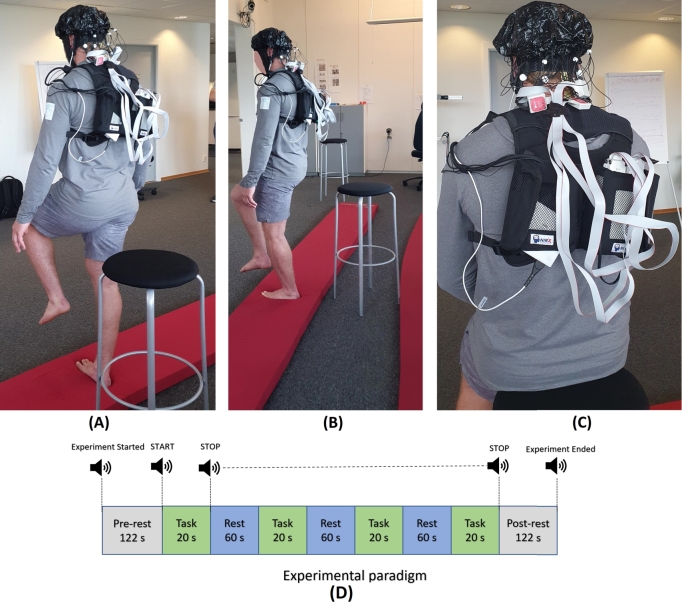
Experimental Setup (A) Left-leg Stance (LLS) (B) Right-leg stance (RLS) (C) NIRSport 2 continuous-wave fNIRS device.

The experiment was conducted in the GAITLINE AS research laboratory in Norway. A continuous-wavelength fNIRS device NIRSport 2 (NIRx Medizintechnik GmbH, Germany), was used to collect the data at the rate of 12.209 Hz as shown in Fig. 1C. The experimental paradigm consisted of 122 seconds of pre-rest and post-rest, a task period of 20 seconds (repeated four times in a single run), and an in-between rest period of 60 seconds (see Fig. 1D). A high stool was placed behind the participant to support their weight during the rest period, as shown in Fig. 1A-1B. Three separate tasks were performed: a straight barefooted walk (BFW) on a plan surface, RLS, and LLS on Kybun mat®. During SLS the participant was asked to lift the leg up to knee height. Kybun mat features a rebounding effect that forces the body into constant, minute movements to maintain balance and posture. The Kybun mat® is making the SLS even more challenging. A couple of test runs were performed with instructions to the participant before starting the experiment. It was observed that the participant was holding his breath in some instances and also elevated the rib cage, which might indicate poor intrinsic core stabilization. While performing an SLS on either the left or the right leg, inversion of the foot was commonly utilized to stabilize the body. The participant has stable hip control, and no large movement was observed during the SLS. Contralateral thumb flexion and extension movements were observed during SLS, which was an interesting finding, raising the question of whether it was a distinctive feature or a common behavior in a larger population. The experiment was conducted in a silent laboratory environment because external focus can influence cortical activity during a single-leg stance [20].

### 2.3 Montage

**Figure 2:**
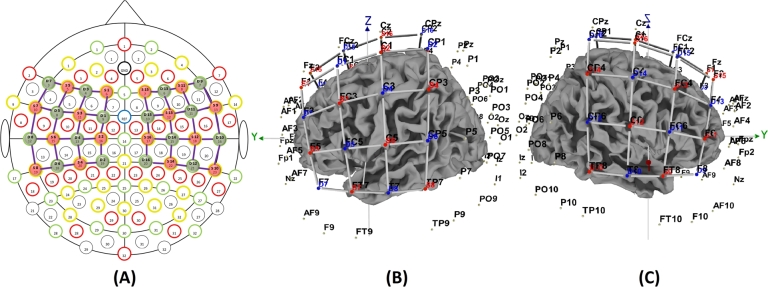
Optodes configuration over the regions-of-interest (ROIs) (A) 2D view (B) 3D view of left hemisphere (C) View of right hemisphere.

Before starting the experiments, the participant's head circumference was measured to ensure the proper selection of NIRxcap. Then, the optodes are placed according to the 10-10 international positioning system over the motor cortex with a 3-*cm* inter-optode distance. Fig. 2 A demonstrates the 2d view of optode arrangement over the motor cortex, while Fig. 2 B and Fig. 2 C shows a 3D view of the left and right sides, respectively. A total number of 16 emitters and detectors were used to collect the data. The Brodmann areas covered by the cerebral cortex are 4, 6, 8, 9, 21, 22, 40, 43, 45, and 48 on the left and right sides of the hemisphere.

### 2.4 Signal prepossessing

**Figure 3:**
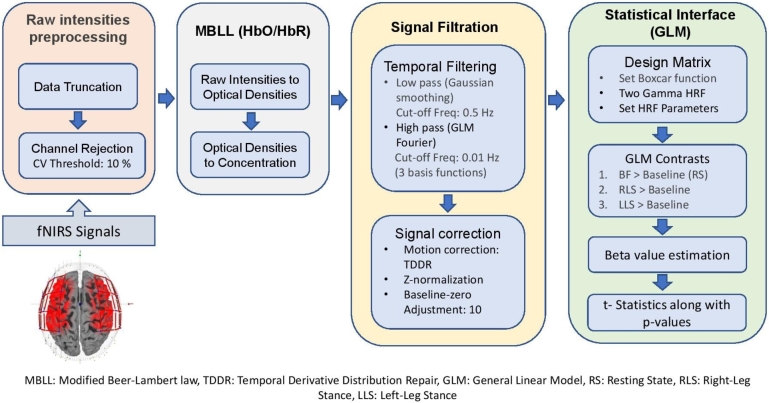
Signal processing pipeline flow diagram.

The signal processing was performed in Satori (Brain Innovation, Germany) and MATLAB® 2021a (The MathWorks Inc., USA). In the first stage, raw intensities channels were rejected by setting the coefficient of variation (CV) with a threshold of 10% [21]. In the second stage, raw intensities were converted into optical densities and then to hemoglobin concentration changes (ΔHbO and ΔHbR) using modified Beer-Lambert law (MBLL) [22]. The temporal derivative distribution repair (TDDR) algorithm was applied to remove baseline shifts and motion spikes from the data [23]. For further correction to the signal baseline zero-adjustments and z-normalization were performed. Fig. 3 shows the signal processing pipeline. After the signal processing, statistical analysis was performed using a general linear model.

### 2.5 General Linear Model (GLM)

A supervised approach for fNIRS called the General Linear Model combines experimental design knowledge and signal morphology from a priori knowledge [24], [25]. In the presence of physiological nuisance signals, this approach provides the best unbiased linear estimate of hemodynamic response to a series of stimuli. GLM serves as a standard statistical method in fNIRS to measure the changes in ΔHbO and ΔHbR. This study analyzes the time series ΔHbO data using GLM with the following model. The simplest form of the model is shown in Eq. (1) [26].

(1) y=Xβ+e

where $X\in R^{N\times M}$ represents the design matrix (*M* denotes the number of time points, and *N* represents the *β* dimension). The $\beta \in R^{M\times L}$ (where *L* is the number of channels) is the corresponding response signal strength for ΔHbO/ΔHbR at the respective *L* channel. The error term is represented by *e*. The GLM fitting procedure finds the set of *β* values that explains the data with a perfect fit. The model's time course values are predicted by the linear combination of predictors provided by the data *y* and design matrix *X* as shown in Eq. (2).

(2) $$\overset{ˆ}{y}=X\beta$$

The prediction of *β* value should be close to *y*, the measured values for a possibly good fit. With the equations rearranged according to the prediction, it becomes apparent that small error values (Eq. (3)) lead to a good prediction.

(3) $$e=y-X\beta =y-\overset{ˆ}{y}$$

It would seem intuitive to find those beta values that would reduce the overall error sum. However, the GLM method does not effectively minimize the sum of error values (Eq. (4)) since errors may contain both positive and negative values. Rather, it finds those beta values that minimize the sum of squared error values.

(4) $$e^{T}e={\left(y-X\beta \right)}^{T}(y-X\beta )\to min$$

The following Eq. (5) gives the optimal beta values (least squares estimates).

(5) $$\beta ={\left(X^{T}X\right)}^{-1}X^{T}Y$$

A *t*-test (Eq. (6)) is used to test the regression coefficient *β* and residual error *e*. The *t*-values are calculated by using the following formula:

(6) $$t=\frac{c^{T}\beta }{\sqrt{e^{2}c^{T}{\left(X^{T}X\right)}^{-1}c)}}$$

## 3 Results and discussion

**Figure 4:**
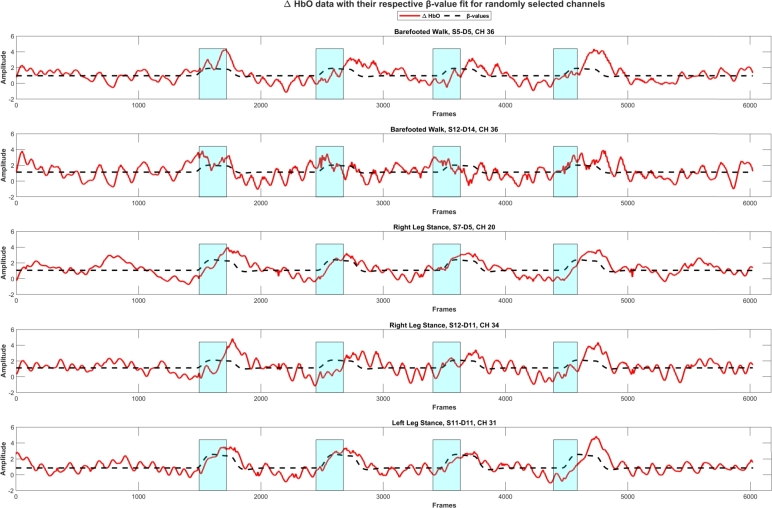
Demonstration of optimized HRF fit (dotted line) to the Δ*HbO* signal (red line).

The GLM is being applied to the fNIRS signal as a supervised approach to recover hemodynamic responses concerning different task conditions across all the trials. The model fit (predicted hemodynamic response) and the randomly selected ΔHbO signal for different conditions and channels are shown in Fig. 4. The highlighted region shows the task duration. The GLM is applied to the individual condition (four trials per condition) of a BFW, LLS, and RLS. In the current study, we only considered ΔHbO. A threshold of *p*-value <0.05 was set to deem channels as statistically significant active channels. Fig. 6 shows the activation map (*t*-value) of significantly active channels (ΔHbO increase) during the task period compared to the baseline (rest) condition. The color bar in Fig. 6 shows the magnitude (*t*-values) of the ΔHbO changes.

### 3.1 Channels cross-correlation

**Figure 5:**
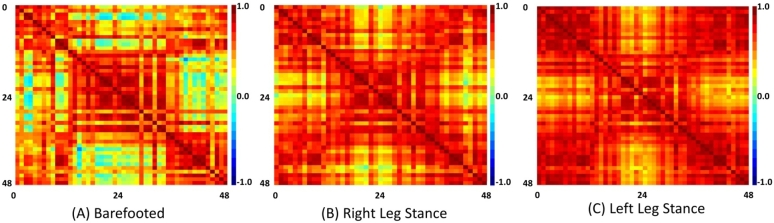
Cross-correlation matrix among channels (0-48) of Δ*HbO* for all three conditions.

The cross-correlation for ΔHbO data was performed individually across all the channels for each condition. The analysis helped find the dominant region and estimate the channel relationship, as shown in Fig. 5. All regions-of-interest (ROIs) were highly correlated in ΔHbO. The highest correlation for ΔHbO data was found in the LLS condition. The temporal regions channels (middle and Broca's areas) from both sides were comparatively less correlated, particularly in walking conditions. Fig. 5 (A-C) shows the cross-correlation heatmap across the channels for barefooted, RLS, and LLS, respectively.

### 3.2 Barefooted walk (BFW)

**Figure 6:**
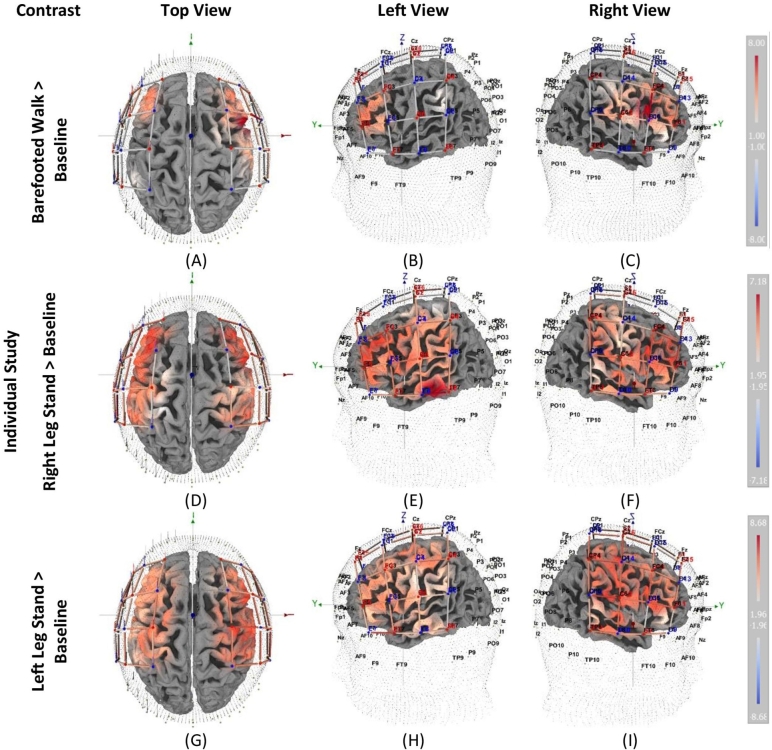
Activation map (*t*-value) for Δ*HbO* based on individual trails.

The BFW is the most natural walking pattern with better foot position, bio-mechanics, and balance control. It was performed to observe the bilateral cortical activation in a quiet realistic walking condition. The activation map of ΔHbO for BFW is shown in Fig. 6 (A-C). More brain ROIs were activated comparatively in the right rather than the left hemisphere. The dorsolateral prefrontal cortex (DLPFC) and Broca's temporal lobe were statistically significantly activated in the left hemisphere. The DLPFC is activated during walking for several reasons, including motor planning and attention allocation. The allocation of attention was also logical since the experiment took place in a laboratory environment with limited space. DLPFC was also activated in SLS due to attentional demands for balance. There are diverse connections between DLPFC and various motor regions, including the premotor (PMA), the supplementary motor area (SMA), the cerebellum, and the basal ganglia. Broca's temporal lobe was equally activated in both hemispheres [27]. Pre-Motor (PMA) and SMA are only activated in the right hemisphere. Curiously, no significant activation in the primary motor cortex (M1) was observed on both sides. The possible reason could be that simple BFW, the common and natural way of walking, required less planning and executory movement control than complex walks.

### 3.3 Right-leg stance (RLS)

The right leg is predominantly used during the walk and many sports activities [28], [29]. The ROIs in the right hemisphere are highly activated compared to the left during the RLS, as shown in Fig. 6 (D-F). The middle temporal gyrus (MTG) was the only area highly activated in both hemispheres. It is mainly responsible for language and semantics, visual perception, multi-modal sensory integration, and probably some connection with the prefrontal due to decision and awareness [30]. A sound stimulus was used to ‘START’ and ‘STOP’ the SLS task. It might be the reason for higher activation in the region due to attentive listening of the stimulus sound. Also, the SLS is a challenging task in addition to the Kybun mat, making it more difficult to maintain balance. So the participant concentrated on listening to the ‘STOP’ sound. Only a couple of the channels in the M1, Wernicke's area, PMA, and SMA were activated in the left hemisphere in the motor cortex. On the other side, all ROIs in the right hemisphere were activated. The highly activated region in the right hemisphere was the primary motor cortex. The activation across the areas of the motor cortex (PMC, SMA, and M1) is very obvious as they are highly involved areas taking inputs from sensory pathways to generate motor control [31]. The asymmetry motor cortex is excitably reported in the literature [32]. However, most studies showed higher activation in the left motor cortex rather than the right. In our study, we found higher activation in the right motor cortex, which is interesting to observe if the activation reduces after physiotherapy exercise.

### 3.4 Left-leg stance (LLS)

The left leg is considered the strongest support, especially in a right-lateral dominant population. This explains why most people (left-leg jumpers) take the first step up a flight of stairs with their right foot; they are more stable on the left [28]. During the LLS, the ROIs in both hemispheres were significantly activated, as shown in Fig. 6(G-I). However, the highest activation (higher *t*-values) occurred at the right hemisphere's PMC, PMA, and SMA. Right-leg dominance (kicking a ball with the right leg) is very common. Studies suggested that there might not be a big difference in balance control in a static position, but in dynamic conditions, the right leg gives better postural stability [33]. Hence, the Kybun Mat continuous rebound effect causes instability and challenges the balance; it is comparable to the dynamics state. It might be the reason for higher activation in ROIs during LLS compared to RLS.

### 3.5 Overall findings

Over the task period, all conditions showed an increase in ΔHbO. Across all channels and conditions, there was a positive cross-correlation. All the ROIs were highly correlated for ΔHbO data. During all three conditions, the ROIs of the right hemisphere were more activated than those of the left hemisphere. In the case of an SLS, an increase in ΔHbO in the prefrontal regions results from surface instability and postural sway; higher attention demands or error processing may be necessary to maintain equilibrium. Significant changes of ΔHbO were found in the motor cortex regions, particularly in SLS. As discussed in the previous sections, activation in the temporal region was observed on both sides and in the SLS conditions. One of the possible reasons for this might be the underlying anatomical peculiarities (presence of extracranial vessels) [21]. Maintaining balance on the right leg is comparatively easier than on the left for a right-legged person. The results also support our hypothesis of increased oxygen demands during an unstable condition (LLS) compared to a stable one (RLS or barefooted condition). Although the result was based on an individual participant, it gives a foundation for further experimentation in the future with a larger population, particularly athletes such as alpine skiers. The participant got an injury a few years ago on his left leg and recovered after many exercises and physiotherapy sessions. Nevertheless, we focus on specific physiotherapy sessions that can enhance balance control on both sides and reduce cortical activation. The results presented in this work are post-physiotherapy. However, further investigation will be planned when the participant completes various physiotherapy sessions designed to enhance balance and control on the left side.

## 4 Conclusion

Compared to the barefooted walk and right-left stance, the participant struggled with balance during the left-leg stance. Results indicate that the right hemisphere experiences higher ΔHbO changes than the contralateral side, particularly during left-leg stance, suggesting enhanced stability demand attention, motor, and sensory function. It provides a foundation for further experimentation with alpine skiers, where balance is of utmost importance on both sides. To improve balance in less time and more efficiently, finding the relationship between brain activation and specific physiotherapy exercise might be an interesting way forward, particularly in rehabilitation and sports.

## Author contribution statement

**Haroon Khan:** Conceived and designed the experiments; Performed the experiments; Analyzed and interpreted the data; Contributed reagents, materials, analysis tools or data; Wrote the paper. **Nauman Khalid Qureshi:** Analyzed and interpreted the data; Contributed reagents, materials, analysis tools or data. **Anis Yazidi:** Analyzed and interpreted the data; Contributed reagents, materials, analysis tools or data. **Håvard Engell:** Performed the experiments; Contributed reagents, materials, analysis tools or data. **Peyman Mirtaheri:** Conceived and designed the experiments.

## Funding statement

This research did not receive any specific grant from funding agencies in the public, commercial, or not-for-profit sectors.

## Data availability statement

Data will be made available on request.

## Declaration of interest's statement

The authors declare no competing interests.
